# Myeloperoxidase-DNA complex: a marker and combined target for *Pseudomonas aeruginosa*-associated bronchiectasis

**DOI:** 10.1186/s13568-026-02012-w

**Published:** 2026-01-22

**Authors:** Shaochu Zheng, Jinling Tang, Xiaopu Wu, Cao Qing, Yun Jiang, Wei Lu, Chongxi Bao, Kangkang Hong, Jing Luo, Jinliang Kong

**Affiliations:** 1 Department of Pulmonary and Critical Care Medicine, The First Affiliated Hospital of Guangxi Medical University, Nanning, Guangxi China; 2 Department of Geriatric Medicine, The Fourth Affiliated Hospital of Guangxi Medical University, Liuzhou, Guangxi China

**Keywords:** Neutrophil extracellular trap (NETs), MPO-DNA complex, Bronchiectasis, Chronic respiratory diseases (CRDs), Pseudomonas aeruginosa

## Abstract

*Pseudomonas aeruginosa* (*P. aeruginosa*)-associated bronchiectasis remains a clinical challenge due to lacking diagnostic biomarkers and targeted therapies. This multi-dimensional (clinical-cell-animal) study investigated the myeloperoxidase (MPO)-DNA complex, a marker of neutrophil extracellular traps (NETs), and the combination of AZD5904 (an MPO inhibitor) and DNase I (a NETs-DNA degrader). Key findings include: First, integrated evidence from bronchoalveolar lavage fluid analysis and immunofluorescence identified the MPO-DNA complex as a biomarker significantly associated with *P. aeruginosa*-associated bronchiectasis severity (e.g., lung lobe involvement, Bronchiectasis Severity Index). Mendelian randomization (MR) analysis revealed a potential causal link with bronchiectasis risk but not with several other chronic respiratory diseases. Second, in cellular models, *P. aeruginosa* PAO1-induced NETs were associated with epithelial damage, as evidenced by the upregulation of reactive oxygen species, malondialdehyde, interleukin-1β, and interleukin-6, and the reduction of BEAS-2B cell viability. Third, in *P. aeruginosa*-infected bronchiectasis rat models, AZD5904 combined with DNase I alleviated lung pathology, inflammation, and NETs accumulation more effectively than monotherapy. This study suggests the MPO-DNA complex as a potential biomarker and pathogenic factor associated with disease severity, and proposes a dual-target combined intervention strategy worthy of further preclinical investigation for *P. aeruginosa*-associated bronchiectasis.

**Supplementary Information:**

The online version contains supplementary material available at 10.1186/s13568-026-02012-w.

## Introduction

Neutrophils play a central role in immune defense. They exert their effects primarily through three pathways: phagocytosis, degranulation, and the release of neutrophil extracellular traps (NETs). NETs are complex extracellular meshwork structures that decondense chromatin, consisting of granule proteins, such as histones, cytoplasmic, elastase enzymes, myeloperoxidase (MPO), etc. (Brinkmann et al. 2004). Typically, upon pathogen stimulation, neutrophils release NETs to “trap” and inhibit their spread, exerting neutralization and killing effects on the microbes through internal components. However, excessive release of NETs can also lead to tissue damage, promoting the development of diseases, such as tumors (Chu et al. 2021), sepsis (Zhang et al. 2023), respiratory diseases, etc. (Keir and Chalmers 2022).

Chronic respiratory diseases (CRDs), such as bronchiectasis, chronic obstructive pulmonary disease (COPD), chronic bronchitis, idiopathic pulmonary fibrosis (IPF), and asthma share a common feature of airway inflammation. Several studies have suggested that NET formation may be associated with severity of chronic airway inflammatory diseases, frequency of acute attacks, and disease progression (Keir and Chalmers 2022). However, considering the presence of confounding factors and reverse causality in previous studies, the causal relationship between exposure factors and outcomes may be overestimated or underestimated. Therefore, the causality between NETs and CRDs cannot be fully determined and requires further validation. Hence, Mendelian randomization (MR) studies can overcome the aforementioned limitations and provide credible evidence of causality (Birney 2022; Richmond and Davey 2022).

This study systematically investigated the role and targeted intervention value of the NETs marker, MPO-DNA complex, in chronic respiratory diseases, particularly in bronchiectasis associated with *P. aeruginosa* infection, using a three-tiered research design encompassing Mendelian randomization analysis, clinical sample validation, and cellular and animal experimental interventions.

## Methods

### Data collection

The GWAS dataset for the NET biomarker MPO-DNA complex was obtained from Gudjonsson et al.’s findings (Gudjonsson et al. 2022), which involved 5357 participants who aged 66–96 years from the AGES-Reykjavik study. GWAS data are also available through the “GWAS Catalog” public database (GCST90087967). GWAS datasets for CRDs were obtained from “FinnGen Release 9” (Date of release: December 18, 2023), including 2188 cases of bronchiectasis (311286 controls), 18,266 cases of COPD (311286 controls), 1150 cases of chronic bronchitis (311286 controls), 34,343 cases of asthma (202399 controls), and 2018 cases of IPF (373064 controls), and further details are recorded on the “FinnGen” website (Kurki et al. 2023). Additionally, another GWAS dataset for bronchiectasis (GCST90044075) was downloaded from the “GWAS Catalog” database for validation, comprising 583 cases of bronchiectasis and 455,765 controls (Jiang et al. 2021). A summary of the download process for GWAS data pertaining to the 5 CRDs is presented in Table 1.

**Table 1:** Download information for GWAS data of five chronic respiratory diseases

Trait	Year	First author	Population	Consortium	Number of cases	Number of controls
Bronchiectasis	2023	-	European	FinnGen	2188	311,286
COPD	2023	-	European	FinnGen	18,266	311,286
IPF	2023	-	European	FinnGen	2018	373,064
Chronic bronchitis	2023	-	European	FinnGen	1150	311,286
Asthma	2023	-	European	FinnGen	34,343	202,399
Bronchiectasis	2021	Jiang, L	European	GCST90044075	583	455,765

### Patients

Bronchoalveolar lavage fluid (BALF) samples were collected from patients with *P. aeruginosa* - infected bronchiectasis who underwent bronchoscopy at the First Affiliated Hospital of Guangxi Medical University between July 2021 and December 2022, prior to any treatment. Concurrently, the clinical data of these patients were collected.

The inclusion criteria for patients with *P. aeruginosa* - infected bronchiectasis were as follows:

1. The diagnosis of bronchiectasis conformed to the relevant diagnostic criteria specified in the Expert Consensus on the Diagnosis and Treatment of Adult Bronchiectasis, and was confirmed by combining clinical symptoms with high - resolution computed tomography of the chest.
2. Patients were aged over 18 years.
3. *Pseudomonas aeruginosa* (*P. aeruginosa*) infection was confirmed by next - generation sequencing or sputum culture.

The exclusion criteria were:

4. Presence of immune system diseases.
5. Comorbidity with acquired immunodeficiency syndrome (AIDS) and other autoimmune diseases.
6. Comorbidity with diseases that may affect the study results, including lung malignancies, active pulmonary tuberculosis, COPD, and bronchial asthma.
7. Comorbidity with severe dysfunction of major systems such as the heart, brain, and kidneys, as well as patients who were unable to cooperate with the designated study procedures.

Control subjects were also required to meet the above - mentioned exclusion criteria and be non - bronchiectasis or non - *P. aeruginosa* - infected individuals. Written informed consent was obtained from all participants, and the study protocol was approved by the Ethics Committee of the First Affiliated Hospital of Guangxi Medical University.

### Preparation of Sprague-Dawley (SD) rats

Thirty SD rats (half male and half female, 6–8 weeks old, weighing 200–220 g) were purchased from the Animal Experimental Center of Guangxi Medical University. All rats were housed in a specific pathogen-free (SPF) animal facility and acclimated to the environment for at least 3 days before model establishment. The housing conditions were as follows: temperature of (20 ± 2) °C, humidity of 50%, ad libitum access to food and water, and a 12-hour light/12-hour dark photoperiod. The use of experimental animals complied with the National Regulations for the Administration of Experimental Animals, and the study was approved by the Animal Ethics Committee of Guangxi Medical University.

### MR analysis between MPO-DNA complex and CRDs

- The MR analysis involves several key steps. Initially, IV selection is performed, where the MPO-DNA complex is designated as the exposure factor, and SNPs exhibiting strong correlations with the MPO-DNA complex are selected as IVs.
- The criteria for IV selection are summarized as follows: 1. SNPs significantly associated with the MPO-DNA complex at a genome-wide level (P < 5 × 10^− 8^). 2. Ensuring no apparent linkage disequilibrium (LD) between SNPs, with an LD coefficient r2 < 0.001 and LD region width > 10,000 kb to maintain SNP independence. Additionally, individual screening of SNPs using the “PhenoScanner” website to exclude pleiotropy effects on outcomes. 3. Merging SNPs with outcome factors and removal of SNPs significantly associated with outcomes at a genome-wide level (P < 5 × 10^− 8^). 4. Harmonizing exposure and outcome result datasets, removing SNPs with palindromic sequences, and conducting MR-PRESSO test to eliminate outlier SNPs. 5. Calculating the F-statistic to assess the strength of genetic variation, with an F-value > 10, indicating strongly correlated IVs, and removing weakly correlated IVs. Subsequently, the MR analysis proceeds as follows: 1. Utilizing the inverse variance-weighted (IVW) method as the primary analysis approach, supplemented by MR Egger, weighted median (WM), weighted model, and simple model as additional MR methods. 2. Expressing the estimated causal effects of MR analysis as odds ratios (ORs) and their 95% confidence intervals (95% CIs). A P-value cutoff of 0.05 is employed to determine causality. 3. Conducting the analysis using the “TwoSampleMR” R package. Finally, sensitivity analysis involves: 1. Employing Cochran’s Q test to assess heterogeneity, with a Q-value greater than 0.05 indicating no heterogeneity. 2. Using the MR Egger regression intercept test to evaluate potential pleiotropy, with a P-value > 0.05 indicating no horizontal pleiotropy of IVs. The “leave-one-out” test was conducted to indicate whether the results would change after removing each SNP. A substantial alteration in the effect suggested that a particular SNP could significantly influence the outcome, highlighting the robustness of the original MR analysis result.

### Culture of *P. aeruginosa* strain and preparation of bacterial suspension

The resuscitated *P. aeruginosa* PAO1 strain (ATCC) from a single colony was inoculated onto agar medium and cultured for 16 h at 37 °C with shaking at 150 rpm. The bacterial pellet was collected by centrifugation and resuspended in broth. The bacterial concentration was determined using the McFarland turbidity method and adjusted to 1 × 10^8^ colony-forming units (CFU)/mL.

### Acquisition of NETs

Neutrophils were isolated from the peripheral blood of patients diagnosed with bronchiectasis using a human neutrophil isolation kit (TBD, Tianjin, China) according to the manufacturer’s protocol. Subsequently, the isolated neutrophils were resuspended in 2 ml Roswell Park Memorial Institute (RPMI) 1640 medium and seeded into 6-well plates at a cell density of 1 × 10^6^ cells/mL. Neutrophils in the PAO1 group were stimulated with 200 µL of PAO1 bacterial suspension (1 × 10^8^ CFU/mL), where as those in the LB group were treated with an equal volume of LB broth. Both groups were incubated for 4 h at 37 °C in a humidified atmosphere containing 5% CO_2_. After incubation, the supernatant was discarded from both the PAO1 group and the LB group. The remaining adherent material in the PAO1 group (with enriched soluble NETs) was gently rinsed with Dulbecco’s Modified Eagle Medium (DMEM), and the same rinsing procedure was performed in the LB group (with negligible soluble NETs). The samples were centrifuged at 450×g for 5 min at 4 °C to obtain a cell-free supernatant, which was then used for subsequent intervention experiments with BEAS-2B cells. The preparation from the PAO1 group is referred to as “NETs” in the cellular assays. To quantify the NETs content, the concentrations of NETs-DNA and MPO in both suspensions were quantified by PicoGreen assay and enzyme-linked immunosorbent assay (ELISA), respectively, as described below.

### Quantification of NETs-DNA content by PicoGreen assay

NETs-DNA was quantified using the Quant-iT PicoGreen dsDNA Quantitation Kit (Yeasen, Shanghai, China). The PicoGreen dsDNA quantitation reagent was diluted 1:200 with 1×TE buffer to prepare 1xPicoGreen stain. the dsDNA standard was serially diluted with 1×TE buffer to prepare a series of concentration gradients, and both the standards and samples were added to the wells of a microplate. A total of 100 µL of 1×PicoGreen stain stain was added to each well, and the microplate was incubated in the dark for 5 min. Subsequently, the fluorescence intensities of the standards and test samples were measured using a fluorescence microplate reader with the excitation wavelength set at 480 nm and the emission wavelength set at 520 nm.

### Correlation analysis of MPO concentrations

The ELISA was used to detect MPO concentrations in the BALF of clinical patients, supernatants of isolated NETs, and BALF of rats. A total of 100 µL of standards and supernatants of test samples were added to the wells of the microplate, and subsequent steps were carried out strictly following the manufacturer’s instructions. The absorbance was measured at a wavelength of 450 nm, and MPO concentrations were quantified based on the standard curve.

Correlation analyses were performed to evaluate the relationships between MPO concentration and the following variables in bronchiectasis patients: NETs-DNA concentration, age, gender, body mass index (BMI), smoking history, duration of bronchiectasis, number of acute exacerbations in the past year, number of involved lung lobes, Bronchiectasis Severity Index (BSI), and Bronchiectasis Aetiology and Co-morbidity index (BACI).

### Immunofluorescence staining for NETs

Pretreatment of Clinical BALF Samples: Collected clinical BALF samples were centrifuged at 300×g for 10 min at 4 °C. The pellet was resuspended in 500 µL of phosphate-buffered saline (PBS), and the suspension was added to a 24-well cell culture plate pre-loaded with cell climbing slices. The plate was incubated in a cell culture incubator at 37 °C with 5% CO_2_ for 1 h to allow cells in the BALF to sediment and adhere to the cell climbing slices. Pretreatment of Neutrophil Suspensions: Sterile cell climbing slices were placed in a 24-well plate, and neutrophil suspensions were added for incubation in the incubator for 30 min. Subsequently, 200 µL of PAO1 bacterial suspension at a concentration of 1 × 10^8^ CFU/mL was added to the wells for continuous stimulation for 4 h. Immunofluorescence Staining Procedure: After incubation, both types of samples were fixed with 4% paraformaldehyde, permeabilized with 0.2% Triton X-100, and blocked with 10% goat serum. Primary antibodies against myeloperoxidase (MPO, 1:400 dilution, Abcam) and citrullinated histone H3 (CitH3, 1:400 dilution, Abcam) were added, followed by overnight incubation at 4 °C. The next day, primary antibodies were recovered, and corresponding secondary antibodies were added. Cell nuclei were stained with 4’,6-diamidino-2-phenylindole (DAPI), and the glass slides were inverted and mounted on slides containing anti-fluorescence quencher. Observations were performed using an immunofluorescence microscope.

### Culture and grouping of BEAS-2B cells

To evaluate the effect of NETs stimulation on airway epithelial cells, subsequent experiments were performed using the human bronchial epithelial cell line BEAS-2B (Procell, Wuhan, China). BEAS-2B cells were cultured in DMEM supplemented with 10% fetal bovine serum (FBS; Gibco, USA) and 1% penicillin-streptomycin (100 U/mL penicillin, 100 µg/mL streptomycin). The cells were maintained in a humidified incubator at 37 °C with 5% CO_2_. Based on the previously prepared supernatant samples, bronchial epithelial cell intervention experiments were performed: 200 µL of supernatant derived from the LB group was added to each well of the control group, while 200 µL of supernatant derived from the PAO1 group was added to each well of the NETs group. DMEM complete medium containing 10% FBS and 1% penicillin-streptomycin was added to both groups to adjust the final volume to 2 mL per well. After 24 h of intervention, samples were collected for subsequent assay of relevant indicators.

### Animal modeling and grouping

Sample size justification: The sample size for animal experiments (*n* = 6 per group) was determined based on preliminary data and power analysis using G*Power 3.1 software, aiming to detect a 30% difference in primary outcome with 80% power and α = 0.05. Thirty SD rats were randomly divided into 5 groups (*n* = 6 each): sham group, *P. aeruginosa*-infected bronchiectasis group (model group), MPO inhibitor group (AZD5904 group), NETs-DNA inhibitor group (DNase I group), and combined inhibitor group (AZD5904 + DNase I group).

The rat model of *P. aeruginosa*-infected bronchiectasis was established by modifying the method described by Wan et al. (Wan et al. 2002), with the following procedures: After tracheotomy, a 1 mL sterile syringe was used to aspirate 0.1 mL of PAO1 bacterial suspension at a concentration of 1 × 10^8^ CFU/mL (for the model group, AZD5904 group, DNase I group, and AZD5904 + DNase I group) or LB broth (for the sham group), which was then injected into the left lower lung of the rats. Starting from Day 1 post-modeling, the AZD5904 group received daily intraperitoneal injection of AZD5904 solution (10 mg/kg); the DNase I group received daily intraperitoneal injection of DNase I solution (5 mg/kg); the AZD5904 + DNase I group received daily concurrent intraperitoneal injections of the above two solutions, for 7 consecutive days. AZD5904 and DNase I were purchased from MedChemExpress (USA). The general condition of the rats was observed daily. Fourteen days later, the rats were anesthetized and sacrificed. Three rats were randomly selected from each group, and their left lungs were harvested for hematoxylin-eosin (HE) staining, followed by pathological scoring. A total of 2 mL of PBS was injected into the left lung via the bronchus using a syringe, followed by repeated aspiration to harvest BALF from the lesioned lung tissue.

### HE staining

Lung tissues were immediately fixed in 10% neutral buffered formalin solution to preserve tissue morphology. After fixation, the tissues were subjected to standard paraffin embedding, which included sequential gradient ethanol dehydration, xylene clearing, and molten paraffin infiltration, followed by embedding. The paraffin blocks were sectioned into 4–5 μm thick slices using a microtome. The slices were attached to poly-L-lysine-coated glass slides to prevent detachment. Prior to HE staining, the sections were dewaxed, rehydrated through a gradient of ethanol, and finally rinsed with distilled water. The standard HE staining protocol was performed as follows: the sections were immersed in Harris hematoxylin for nuclear staining, differentiated with 1% acid alcohol to reduce non-specific staining, then blued with warm tap water, and finally counterstained with eosin for cytoplasm and extracellular matrix. The stained sections were dehydrated, cleared with xylene, and mounted with neutral balsam to prepare permanent sections.

### Tracheobronchial histopathological score

We employed a tracheobronchial histopathological score, evaluating the following features in stained sections: (1) columnar ciliated epithelium, (2) gland cells, (3) intraluminal secretions, and (4) inflammatory cell infiltration. Pathological scoring was performed blindly by two independent pathologists, using a semi-quantitative scale adapted from the method described by Fan et al. (Fan et al. 2016).

### Cell viability assay

The viability of BEAS-2B cells was determined using the Cell Counting Kit-8 (CCK-8) assay. We collect the treated cells and replace the culture supernatant with fresh medium containing 10% CCK-8, and then measured the absorbance at 450 nm using a multi-mode microplate reader to quantify cell viability.

### Reactive oxygen species (ROS) detection

The intracellular ROS level of BEAS-2B cells was detected using an ROS Detection Kit (Beyotime, China). After treatment, the cells were incubated with 1 mL of DCFH-DA fluorescent probe working solution (diluted 1:1000 in serum-free medium) in a 37 °C incubator for 20 min. Subsequently, the fluorescence intensity was analyzed using a flow cytometer to quantify ROS levels.

### Malondialdehyde (MDA) quantification

The relative concentration of MDA in BEAS-2B cells and BALF of lesioned lung tissues from rats was determined using a Lipid Peroxidation Assay Kit (Beyotime Biotechnology, Shanghai, China). MDA reacts with thiobarbituric acid (TBA) in the sample to form an MDA-TBA adduct, and absorbance was measured at 532 nm.

### Detection of inflammatory cytokines

The levels of IL6 and IL1β in BEAS-2B cells supernatants and rat BALF were measured using ELISA. A total of 100 µL of standards and supernatants of test samples were added to the wells of the microplate, with subsequent procedures performed strictly according to the kit instructions. Absorbance was measured at 450 nm, and the concentrations of IL6 and IL1β were calculated using the standard curve.

### Statistical methods

All statistical analyses and data processing were performed using R software, IBM SPSS Statistics 26.0, and GraphPad Prism 9.5. Unpaired t-tests were used for comparisons between two groups, while one-way analysis of variance (ANOVA) with Tukey’s post-hoc test was performed for multiple comparisons. The monotonic correlation between two variables was assessed using Spearman’s rank correlation analysis. Data are expressed as the mean ± standard error of the mean (SEM). A P value < 0.05 was considered statistically significant. For cellular experiments, the sample size was set to three independent replicates (*n* = 3). This sample size was determined according to the consistently low coefficient of variation (CV < 15%) of key outcome measures in preliminary experiments, which ensured > 80% statistical power to detect a 20% intergroup difference at a significance level of α = 0.05.

## Results

### MR analysis of the causal relationship between MPO-DNA complex and CRDs

**Fig. 1:**
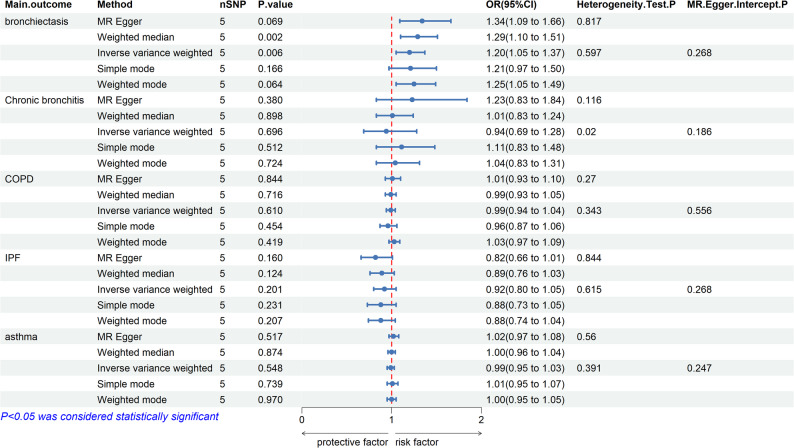
The results of five MR analysis methods to assess the causal relationship between MPO-DNA complex and CRDs

**Fig. 2:**
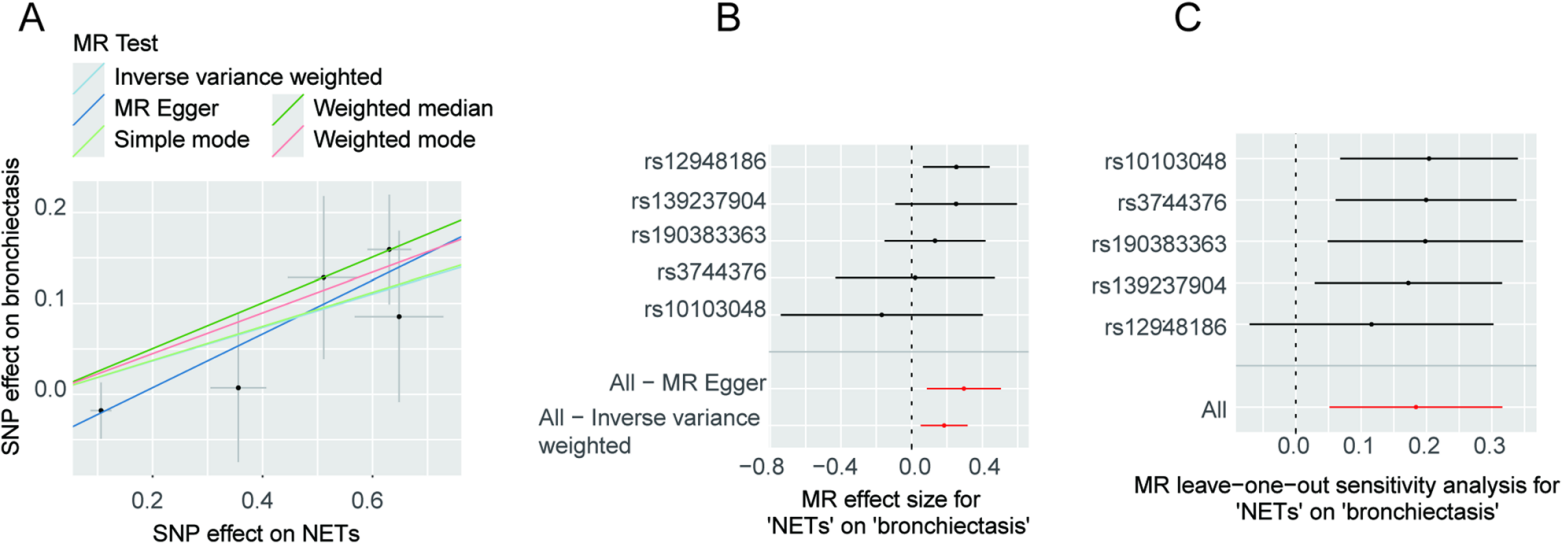
Causal relationship between MPO-DNA complex and bronchiectasis (Finngen). **A** Results of five methods of MR between MPO-DNA complex and bronchiectasis. **B** The effect of each SNP on bronchiectasis and the total effect of MR-Egger method and IVW method. **C** Results of leave-one-out sensitivity test

After filtering according to the abovementioned conditions, five SNPs were considered as IVs for the MPO-DNA complex (Supplementary Table 1). Besides, five MR analysis methods were employed to assess the causal relationship between MPO-DNA complex and CRDs, and detailed analysis results are documented in Supplementary Table 2. The results of the MR analysis were visualized as a forest plot (Fig. 1). Using the IVW method as the primary analysis, genetically predicted higher MPO-DNA complex levels were associated with an increased risk of bronchiectasis (OR = 1.20, 95% CI = 1.05–1.37, *P* = 0.006). Consistent direction of effect was observed with the WM method (OR = 1.29, 95% CI = 1.10–1.51, *P* = 0.002). No significant associations were found between the MPO-DNA complex and COPD, chronic bronchitis, IPF, or asthma (all *P* > 0.05 by IVW). Sensitivity analyses, including leave-one-out, Cochran’s Q test, and MR-Egger intercept test, indicated that the main findings were robust and not driven by single SNPs, heterogeneity, or horizontal pleiotropy (Supplementary Table 3, Fig. 2A-C). In the GCST90044075 dataset, IVW analysis results indicated that MPO-DNA complex could be a risk factor for bronchiectasis (OR = 1.43, 95% CI = 1.01–2.00.01.00, *P* = 0.043), with consistent results obtained by WM analysis (OR = 1.50, 95% CI = 1.06–2.11, *P* = 0.023), while other methods exhibited OR > 1 and *P* > 0.05 (Supplementary Fig. 1A-C). Cochran’s Q heterogeneity test indicated no heterogeneity in IVW (Q = 5.664, *P* = 0.226) and MR-Egger (Q = 4.307, *P* = 0.230) tests. The MR-Egger intercept test did not detect potential horizontal pleiotropy (*P* = 0.403). Additionally, reverse MR analysis was carried out, and it was found that bronchiectasis could not lead to the generation of MPO-DNA complex (Supplementary Table 4).

### Morphological observation of NETs in clinical BALF

**Fig. 3:**
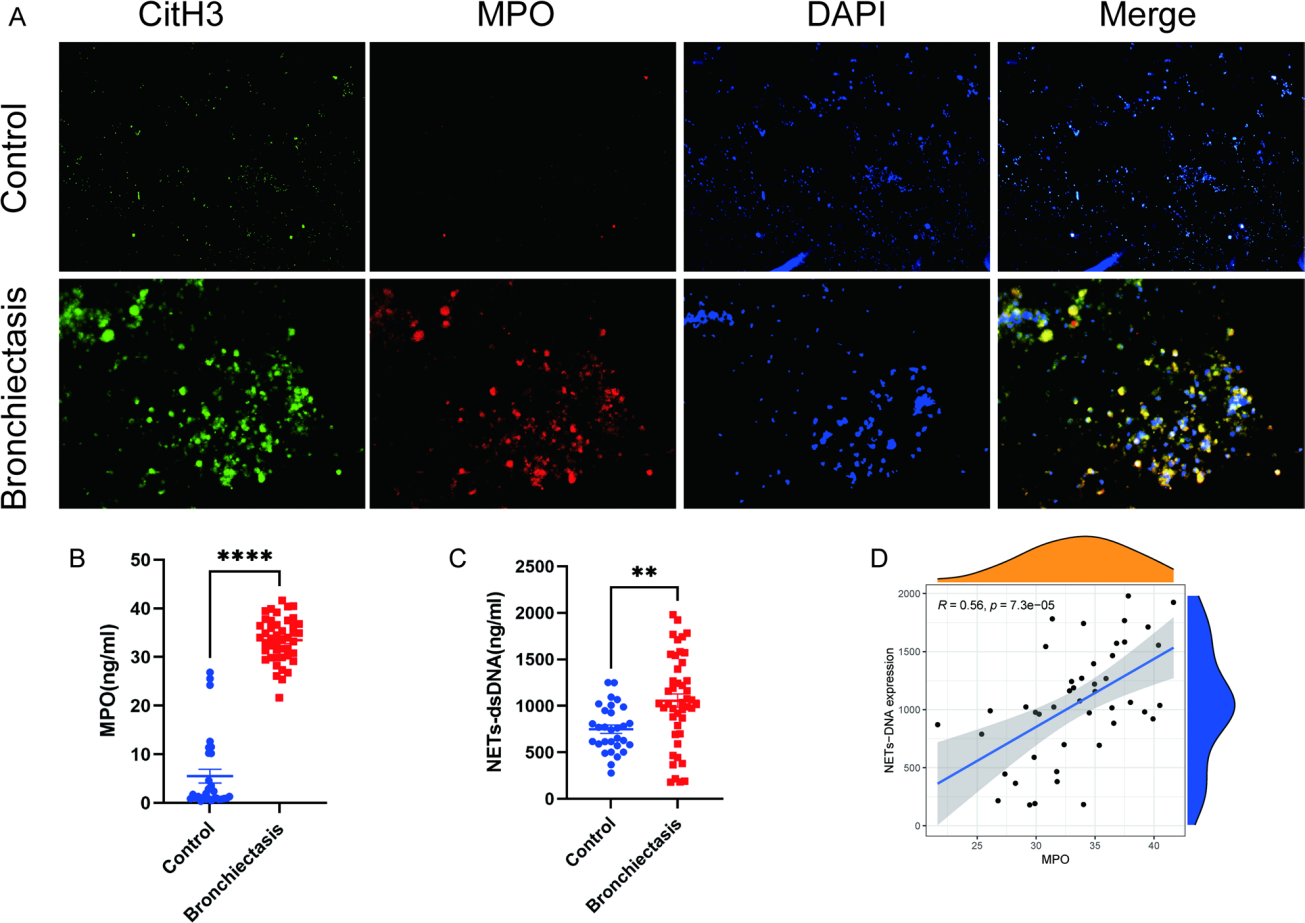
Morphological observation of NETs and analysis of MPO-DNA complex levels in BALF from bronchiectasis patients infected with *P. aeruginosa*. **A** Immunofluorescence co-staining of NETs in BALF from bronchiectasis patients and control group. **B** Comparison of MPO concentrations in BALF between bronchiectasis patients and control group (*n* = 45 vs. *n* = 30, *P* < 0.05). **C** Comparison of NETs-DNA concentrations in BALF between *P. aeruginosa*-infected bronchiectasis patients and control group (*n* = 45 vs. *n* = 30, *P* < 0.05). **D** Correlation analysis between MPO concentrations and NETs-DNA concentrations in BALF of bronchiectasis patients (*r* = 0.56, *P* < 0.001). *P < 0.05, **P < 0.01, ***P < 0.001, ****P < 0.0001, ns: not significant (*P* ≥ 0.05)

Immunofluorescence co-staining revealed that the DNA reticular structures in the BALF of bronchiectasis patients infected with *P. aeruginosa* expressed MPO and CitH3. In contrast, only CitH3 was detected in the cells of the control group, with almost no MPO or CitH3 observed on extracellular structures, and no typical NETs structures were formed (Fig. 3A).

### MPO-DNA levels in clinical BALF

We finally collected BALF samples from 45 bronchiectasis patients and 30 control subjects for MPO-DNA determination. The results showed that the MPO concentrations detected by ELISA in the bronchiectasis group (33.49 ± 0.67 pg/mL) was significantly higher than that in the control group (5.53 ± 1.42 pg/mL) (*P* < 0.05, Fig. 3B). Additionally, the NETs-DNA concentrations quantified by PicoGreen assay in the *P. aeruginosa*-infected bronchiectasis group (1056.00 ± 72.30 pg/mL) was also significantly higher than that in the control group (748.90 ± 45.58 pg/mL) (*P* < 0.05, Fig. 3C). A positive correlation was observed between MPO and NETs-DNA concentrations, with a correlation coefficient of *r* = 0.56 (*P* < 0.001, Fig. 3D).

### Correlation between MPO concentration and clinical data

**Fig. 4:**
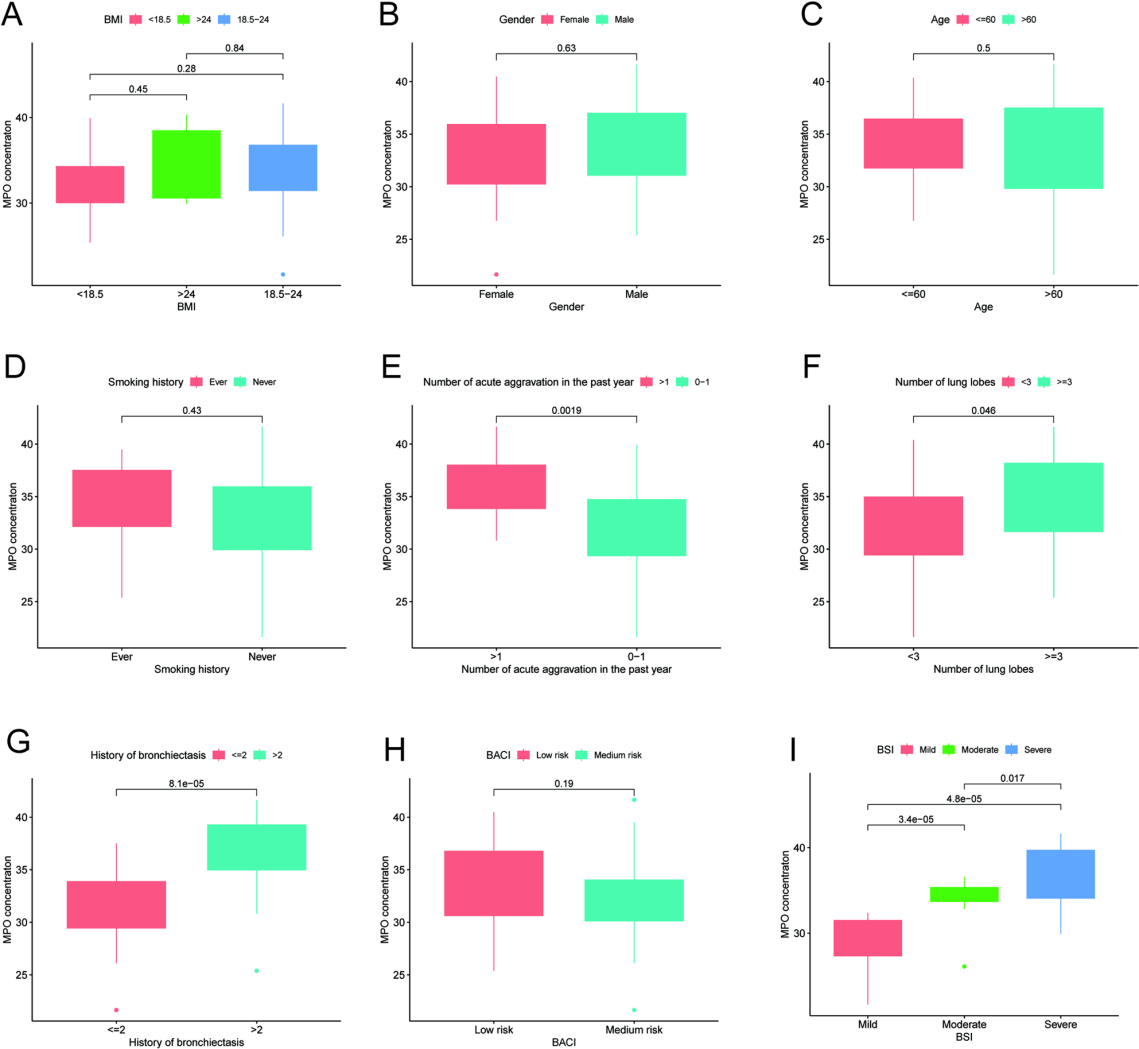
Correlation between MPO concentration in BALF and clinical data of bronchiectasis patients, including BMI (**A**), gender (**B**), age (**C**), smoking history (**D**), the number of acute aggravation in the past year (**E**), number of lung lobes (**F**), history of bronchiectasis (**G**), BACI (**H**) and BSI score (**I**)

The results showed that MPO expression differed among groups stratified by the number of acute exacerbations in the past year, number of involved lung lobes, duration of bronchiectasis, and BSI score. No significant differences in MPO concentration were found among groups stratified by BMI, gender, age, smoking history, or BACI score (Fig. 4A-D, H). Specifically, higher MPO concentrations were observed in the following subgroups: patients with more than one acute exacerbation in the past year, those with involvement of ≥ 3 lung lobes, those with a bronchiectasis duration of more than 2 years, and those with a higher BSI score (Fig. 4E-G, I).

### Acquisition of NETs

**Fig. 5:**
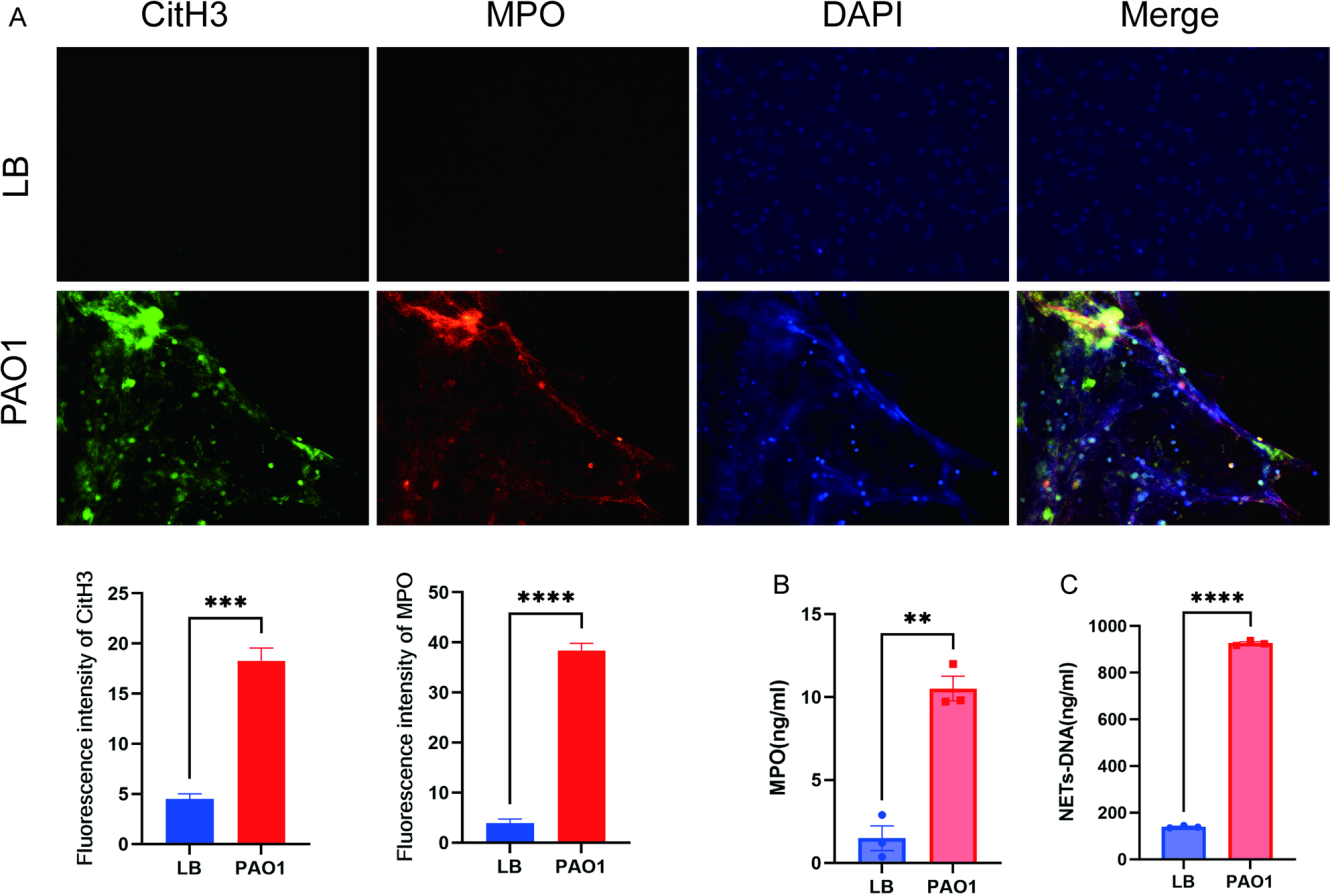
Acquisition of NETs by neutrophils following *P. aeruginosa* PAO1 intervention. **A** Comparison of immunofluorescence co-staining of NETs in neutrophils between PAO1 group and LB group (Red: MPO; Green: CitH3; Blue: DNA, *P* < 0.05). **B** Comparison of MPO concentration in the system between PAO1 group and LB group (*n* = 3, *P* < 0.05). **C** Comparison of NETs-DNA concentration in the system between PAO1 group and LB group (*n* = 3, *P* < 0.05)

Immunofluorescence co-staining demonstrated that neutrophils were capable of generating NETs following stimulation with PAO1. The DNA reticular structures of these NETs were decorated with MPO and CitH3, and the expression levels were significantly higher than those in the LB group (*P* < 0.05, Fig. 5A). Additionally, the MPO concentrations detected by ELISA in the PAO1 group (10.51 ± 0.74 pg/mL) was significantly higher than that in the LB group (1.50 ± 0.74 pg/mL), the NETs-DNA concentrations quantified by PicoGreen assay in the PAO1 group (926.00 ± 6.42 pg/mL) was also significantly higher than that in the LB group (139.30 ± 2.92 pg/mL) (Fig. 5B, C).

### Cell viability

**Fig. 6:**
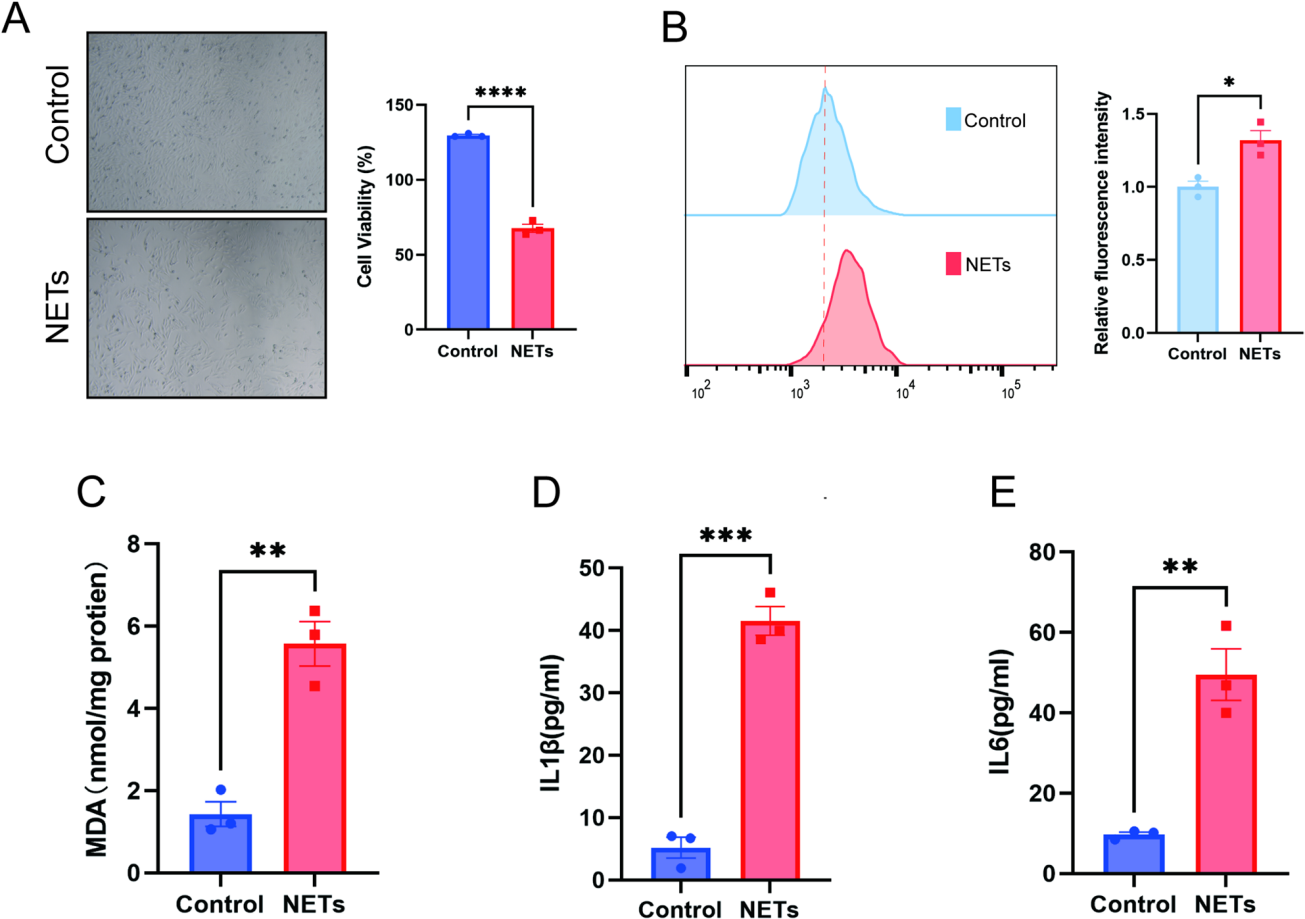
Effects of NETs intervention on viability, inflammation and oxidative stress levels of BEAS-2B cells. **A** CCK-8 assay and microscopic observation under a 10x objective. Comparison of ROS (**B**), MDA (**C**), IL1β (**D**) and IL6 (**E**) in BEAS-2B cells between NETs group and control group (*n* = 3, *P* < 0.05)

CCK-8 assay and microscopic observation under a 10x objective revealed that compared with the control group, the survival rate of BEAS-2B cells in the NETs group was significantly reduced. Specifically, the mean cell viability was 129.6% in the control group and 67.65% in the NETs group (Fig. 6A).

### Inflammation and oxidative stress in BEAS-2B cells

The inflammatory response and oxidative stress of bronchial epithelial cells were evaluated by detecting the levels of ROS, MDA, and inflammatory cytokines in BEAS-2B cells. The results showed that the levels of IL1β, IL6, MDA, and ROS in the NETs-treated group were significantly higher than those in the control group (Fig. 6B–E).

### Pathological features in rat lung tissues

**Fig. 7:**
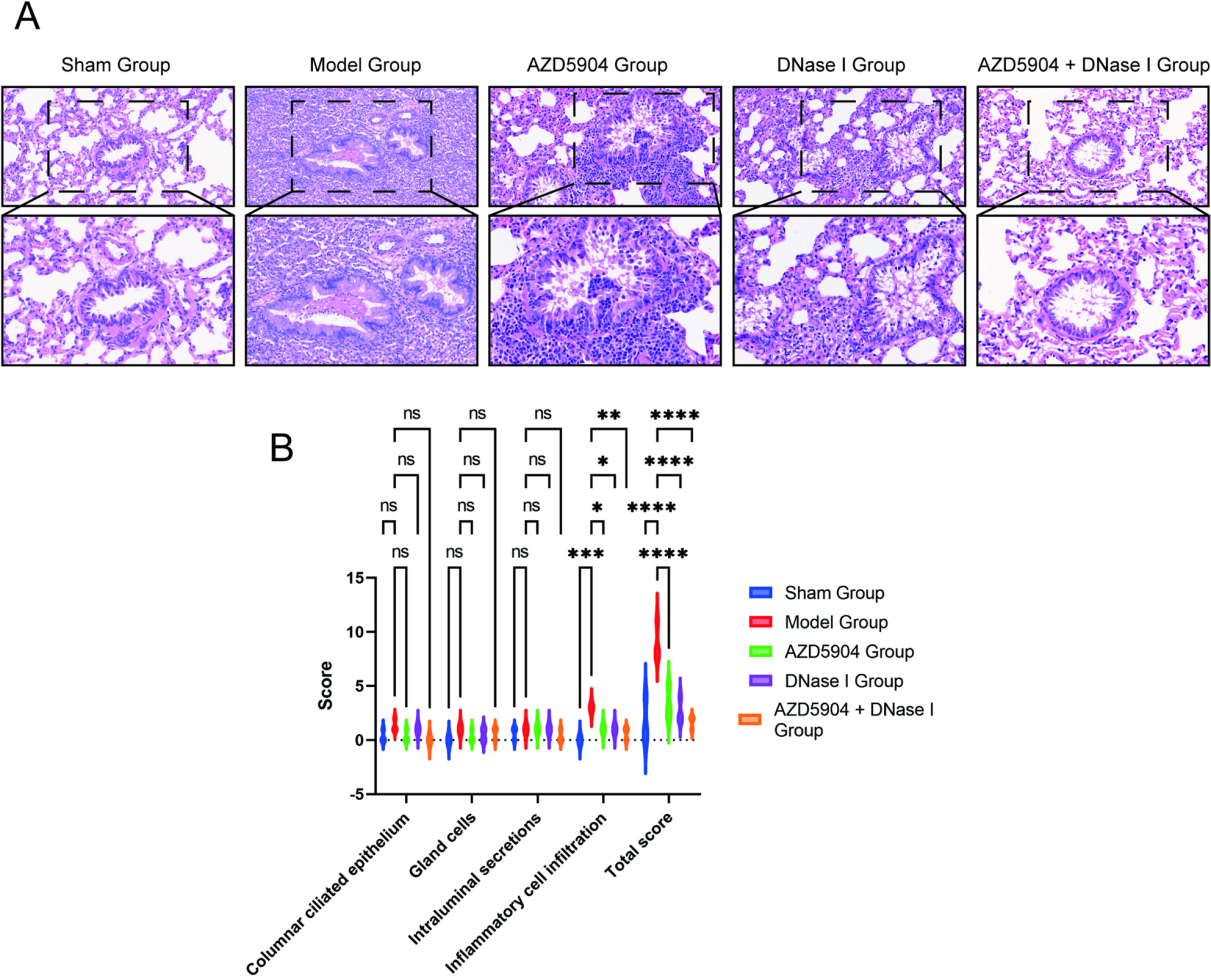
Comparison of pathological characteristics and pathological scores of lung tissue in rat bronchiectasis models among different intervention groups. **A** Observation of pathological morphology of rat lung tissue in different intervention groups (HE staining, 20x objective). **B** Comparison of lung tissue pathological scores among different intervention groups (*n* = 3)

As shown in Fig. 7A, under a light microscope at 20x objective, the tracheobronchial tissue of rats in the sham group exhibited a clear hierarchical structure. The mucosal epithelial cells and cilia were neatly arranged, with no exudate in the lumen. The alveolar tissue structure was clear, without alveolar wall thickening, abnormal infiltration, or hyperplasia. In contrast, rats in the model group showed varying degrees of bronchial wall destruction. The lumen was dilated and filled with pus cells and desquamated epithelium. A large number of inflammatory cells infiltrated the submucosa, muscular layer, and peribronchial lung tissue, and the alveolar tissue structure was obscure. Compared with the model group, the pathological changes in lung tissues of rats in the AZD5904 group and DNase I group were slightly improved, while the improvement was more significant in the AZD5904 + DNase I group.

### Tracheobronchial histopathological score

In terms of tracheal pathological scores, the total score of the model group was significantly higher than that of the sham group. The scores of the AZD5904 group and DNase I group were slightly reduced, while the total score of the AZD5904 + DNase I group was significantly reduced—with the most notable reduction in the inflammatory cell infiltration score (*P* < 0.05, Fig. 7B). The scoring details for each sample were in Supplementary Table 5.

### Detection of MPO-DNA in rats

**Fig. 8:**
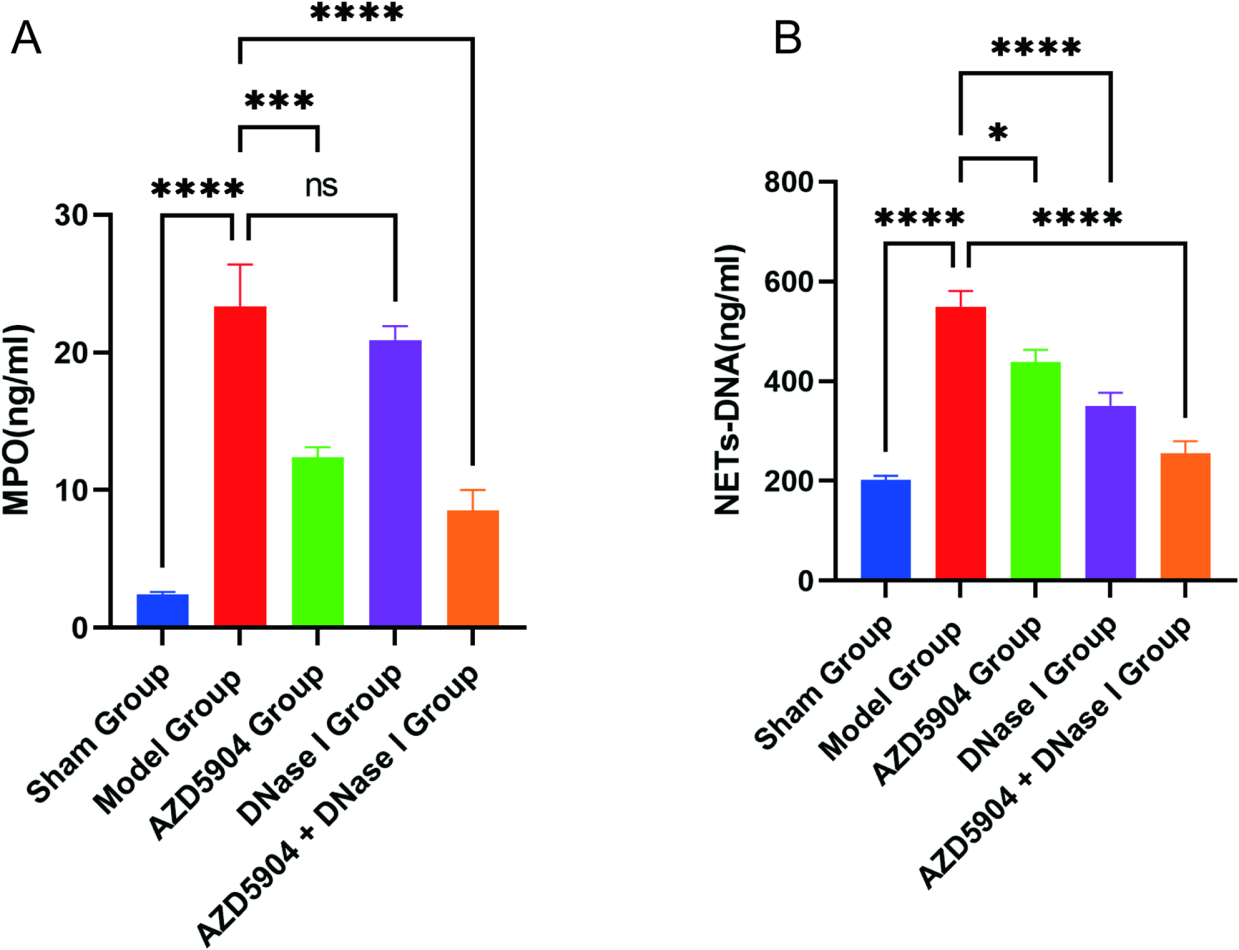
Detection of MPO and NETs-DNA concentrations in BALF of rat bronchiectasis models among different intervention groups. **A** Comparison of MPO concentrations (*P* < 0.05). **B** Comparison of NETs-DNA concentrations. (*n* = 6, *P* < 0.05)

In BALF, the concentrations of MPO and NETs-DNA in the model group were significantly higher than those in the sham group. After intervention with AZD5904 or DNase I, the concentrations of MPO and NETs-DNA decreased accordingly, notably, the combined intervention (AZD5904 + DNase I) exerted a more significant inhibitory effect on the concentrations of MPO and NETs-DNA (*P* < 0.05, Fig. 8A, B).

### Evaluation of pulmonary inflammation in rats

**Fig. 9:**
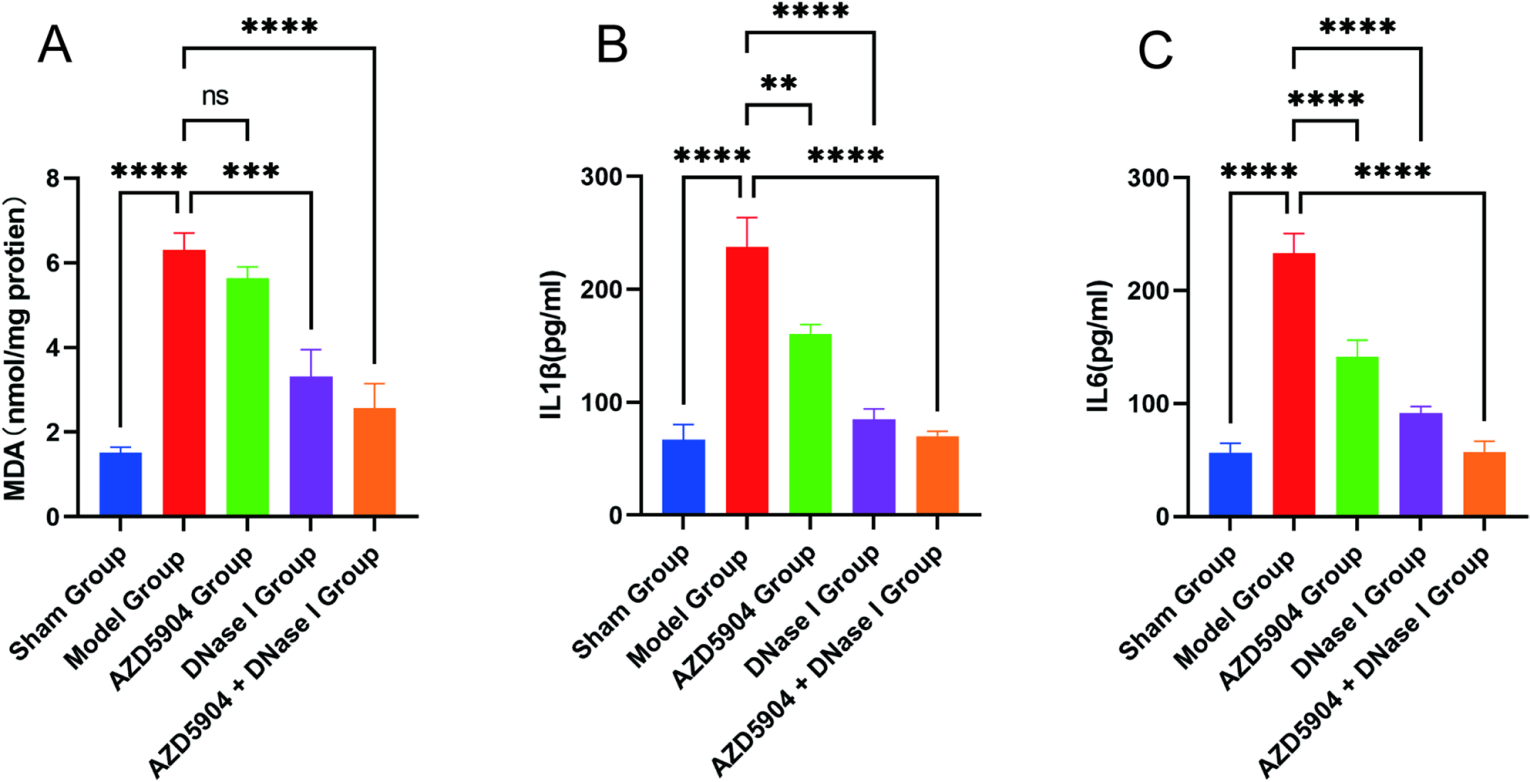
Detection of inflammatory factors (IL1β, IL6) and MDA levels in BALF of rat models among different intervention groups. **A** Comparison of MDA concentrations. **B** Comparison of IL1β concentrations. **C** Comparison of IL6 concentrations. (*n* = 6, *P* < 0.05)

The degree of inflammatory injury in lung tissue was evaluated by detecting the levels of inflammatory cytokines and MDA in rat BALF. The results showed that in BALF, the levels of IL1β, IL6, and MDA in the *P. aeruginosa*-infected bronchiectasis group were significantly higher than those in the sham group. After intervention with DNase I, the concentrations of these inflammatory cytokines and MDA decreased significantly. However, AZD5904 intervention only reduced the levels of inflammatory cytokines (IL1β and IL6) but had no significant effect on MDA levels. Notably, the anti-inflammatory effect of DNase I intervention was better than that of AZD5904, and the combined intervention (AZD5904 + DNase I) exerted a more significant inhibitory effect on the concentrations of inflammatory cytokines and MDA (Fig. 9A–C).

## Discussion

NETs are large mesh-like structures composed of DNA and antimicrobial proteins, capable of capturing pathogens, preventing their dissemination. However, excessive release of NETs can also lead to tissue damage, thereby causing harm to the host. Numerous studies have linked the formation of NETs to CRDs. For instance, studies have shown that double-stranded DNA (dsDNA) released during NETosis plays a critical role in acute exacerbations of COPD. In animal models, treatment with DNase I alleviates virus-induced exacerbation of COPD symptoms (Katsoulis et al. 2024). In severe pulmonary diseases such as acute respiratory distress syndrome, NETs are associated with disease severity and exacerbate tissue damage through direct cytotoxicity, procoagulant activity, and induction of subsequent immune responses (Salzmann et al. 2024). Tissue damage in critically ill COVID-19 patients is closely related to NETs-mediated inflammatory responses (Veras et al. 2020). However, although we know that NETs are closely associated with chronic respiratory diseases, the causal relationship between them remains unclear. Therefore, it is necessary to further investigate the causal relationship between NETs and chronic respiratory diseases.

Our MR study provides genetic evidence supporting a potential causal role of elevated MPO-DNA complex levels in bronchiectasis risk. However, no significant causal association between the MPO-DNA complex and diseases such as COPD, chronic bronchitis, IPF, or asthma was observed. It is important to note that while our MR analysis suggests causal role for bronchiectasis among the CRDs tested, NETs markers like MPO-DNA are known to be elevated in a wide range of infectious and inflammatory conditions. Therefore, its clinical utility as a biomarker likely lies in reflecting disease activity and NETs burden within bronchiectasis, rather than in differential diagnosis from other pulmonary diseases.

Bronchiectasis is characterized by irreversible bronchial dilatation caused by damage to the bronchial wall. The extent of dilatation can range from focal disease confined to a single pulmonary segment or lobe to diffuse disease involving all lung lobes. The clinical manifestations of bronchiectasis patients are highly variable. Some patients are asymptomatic, while most present with symptoms of varying severity, including chronic cough, copious sputum production, and/or intermittent hemoptysis, with or without dyspnea and respiratory failure. Globally, the prevalence of bronchiectasis is on the rise. Current reports indicate a prevalence as high as 566 cases per 100,000 population, representing a 40% increase over the past decade (Flume et al. 2018). Meanwhile, bronchiectasis has diverse etiologies, with severe or recurrent respiratory infections being the most common. Among these infections, *P. aeruginosa* infection and colonization are particularly prevalent, and numerous studies have confirmed their association with worsening lung function and increased mortality (Reynolds and Kollef 2021). Therefore, in the present study, we focused on bronchiectasis associated with *P. aeruginosa* infection.

Our clinical sample detection results further confirmed that the concentrations of MPO and NETs-DNA in the BALF of bronchiectasis patients infected with *P. aeruginosa* were significantly higher than those in the control group, with a positive correlation between the two. Typical NETs structures were observed via immunofluorescence. Correlation analysis revealed that MPO concentration was closely associated with the BSI of bronchiectasis, especially in patients with severe BSI scores, where MPO expression was significantly elevated. These findings indicate that the MPO-DNA complex is associated with disease severity and may serve as a biomarker for monitoring disease activity in *P. aeruginosa*-associated bronchiectasis.

Cellular experiments suggested that NETs induced by *P. aeruginosa* were associated with damage to bronchial epithelial cells. Animal experiment results showed that in the *P. aeruginosa*-induced rat model, NETs accumulation was associated with pulmonary inflammation and oxidative damage. Intervention with either an MPO inhibitor (AZD5904) or a NETs-DNA inhibitor (DNase I) alone mitigated lung pathology and reduced inflammatory and oxidative stress markers; notably, the combined intervention exerted a more pronounced effect. This suggests a potential synergy between inhibiting MPO activity (which may reduce NETs formation or activity) and degrading existing NETs-DNA. These findings provide a preclinical rationale for a novel “dual-target combined intervention” strategy targeting NETs. However, the translational relevance requires careful consideration. Our animal model employs short term bacterial infection, which does not fully recapitulate the chronic cycle of infection, inflammation, and tissue remodeling characteristic of human bronchiectasis. Future studies using chronic infection or colonization models are needed to assess the long-term efficacy and safety of this combined strategy. Furthermore, potential off-target effects of MPO inhibition on host defense and the optimal timing of intervention in the disease course warrant further investigation.

Literature indicates that MPO influences the inflammatory process by regulating neutrophil migration, activation, and NETs formation. Deletion or inhibition of MPO can reduce the infiltration of neutrophils and inflammatory monocytes/macrophages, thereby alleviating inflammation (Ali et al. 2022). The binding of MPO to the neutrophil surface can affect the chemotactic behavior of neutrophils at inflammatory sites (Rehring et al. 2021). The MPO inhibitor IN-4 exhibits dose-dependent inhibition of MPO activity in mouse inflammatory models and may serve as a novel strategy for treating cardiovascular inflammation (Regard et al. 2023). In myocardial infarction models, MPO deficiency or inhibition reduces neutrophil infiltration, thereby mitigating inflammation and myocardial damage (Guthoff et al. 2022; Nettersheim et al. 2023). MPO-deficient mice exhibit milder inflammation and faster recovery in acute/chronic colitis models (Cartwright et al. 2024). Although MPO generally promotes inflammation, in certain contexts (e.g., post-tumor radiotherapy), increased MPO activity may exert anti-tumor effects by inhibiting tumor growth (Ali et al. 2022). Additionally, MPO deficiency may lead to immune dysfunction, increasing the risk of infection and chronic inflammation (e.g., in cases of chronic non-bacterial osteomyelitis) (Sundqvist et al. 2023). Therefore, future studies need to further clarify the spatiotemporal-specific role of MPO in different diseases to optimize therapeutic strategies.

NETs-DNA (dsDNA), the core component of NETs, acts both as an effector molecule in pathological processes and a potential therapeutic target, playing a critical role in various pathophysiological processes. Upon oxidative stress or tissue damage, dsDNA is released into the extracellular space or cytoplasm, where it can activate inflammatory signaling pathways. For instance, extracellular dsDNA drives inflammatory responses by activating the NLRP3 inflammasome and AIM2 inflammasome, thereby promoting the maturation and release of proinflammatory cytokines (e.g., IL1β, IL18) (Lackner et al. 2025; Messaoud-Nacer et al. 2022; Ouyang et al. 2022). In models of Crohn’s disease and colitis, the level of exosome-derived dsDNA is positively correlated with disease activity, and it exacerbates inflammation via the STING pathway (Zhao et al. 2021). Meanwhile, clearing extracellular dsDNA (e.g., via DNase I treatment) or inhibiting NET formation can alleviate inflammatory responses. For example, degrading dsDNA in NETs reduces dsDNA-mediated secondary inflammatory responses in acute lung injury (Messaoud-Nacer et al. 2022), mitigates cardiomyocyte apoptosis (Zhang et al. 2025), and attenuates inflammation in blast-induced lung injury (Meng et al. 2025).

An international, observational, multi-cohort study on bronchiectasis by Keir et al. identified, via sputum proteomics, that NETs-related proteins had the strongest correlation with disease severity. This finding was validated in two observational cohorts: sputum NETs were associated with the BSI, quality of life, future hospitalization risk, and mortality. Furthermore, both macrolide therapy and intravenous antibiotic use during bronchiectasis exacerbations reduced sputum NETs concentrations in patients with bronchiectasis, and a link existed between changes in NETs concentrations and clinical benefits (Keir et al. 2021). These results are consistent with our study findings, revalidating the importance of NETs and suggesting that targeting NETs may serve as a potential mechanistic basis for next-generation bronchiectasis therapies.

It should be noted that this study has certain limitations. First, the MR analysis was based on data from individuals of European ancestry, and the ethnic generalizability of the results needs further validation in Asian populations. Additionally, since the data did not provide covariates, we could not conduct more detailed stratified analyses (e.g., by age, gender, or presence of comorbid infections). Second, the size of the clinical sample was relatively limited, and the diagnostic value of the MPO-DNA complex requires further confirmation with an expanded sample size. Third, animal experiments have not yet explored the downstream molecular mechanisms through which NETs influence bronchiectasis in depth. Fourth, the short term infection model may not fully replicate the chronic pathophysiology of human *P. aeruginosa*-associated bronchiectasis, and the fixed-dose administration immediately after infection in our study differs from the dosing strategies for chronic diseases in clinical practice. The translational potential of the combined intervention requires testing in more clinically relevant models.

Conclusions 

Nevertheless, through multi-dimensional evidence, this study supports an association between the MPO-DNA complex and *P. aeruginosa*-associated bronchiectasis severity and provides preclinical proof-of-concept for a combined therapeutic strategy targeting NETs formation and clearance. This study offers a foundation for further research into the pathogenic mechanisms of bronchiectasis and the development of potential novel therapies.

## Supplementary Information

Below is the link to the electronic supplementary material.

Supplementary Material 1

Supplementary Material 2

Supplementary Material 3

Supplementary Material 4

Supplementary Material 5

Supplementary Material 6

## Data Availability

The data that support the findings of this study are available on request from the corresponding author. The data are not publicly available due to privacy or ethical restrictions.

## Abbreviations

NETs: Neutrophil extracellular traps
MPO: Myeloperoxidase
dsDNA: Double-stranded DNA
BALF: Bronchoalveolar lavage fluid
CRDs: Chronic respiratory diseases
COPD: Chronic obstructive pulmonary disease
IPF: Idiopathic pulmonary fibrosis
MR: Mendelian randomization
BSI: Bronchiectasis severity index
CitH3: Citrullinated histone H3
PAO1: Pseudomonas aeruginosa strain PAO1
ROS: Reactive oxygen species
MDA: Malondialdehyde
DNase I: Deoxyribonuclease I
ELISA: Enzyme-linked immunosorbent assay
