# Supplementary figures and images for: Myeloperoxidase-DNA complex: a marker and combined target for Pseudomonas aeruginosa-associated bronchiectasis
Source: AMB Express. 2026 Jan 22;16:17. doi: 10.1186/s13568-026-02012-w (PMC12909637; doi:10.1186/s13568-026-02012-w)

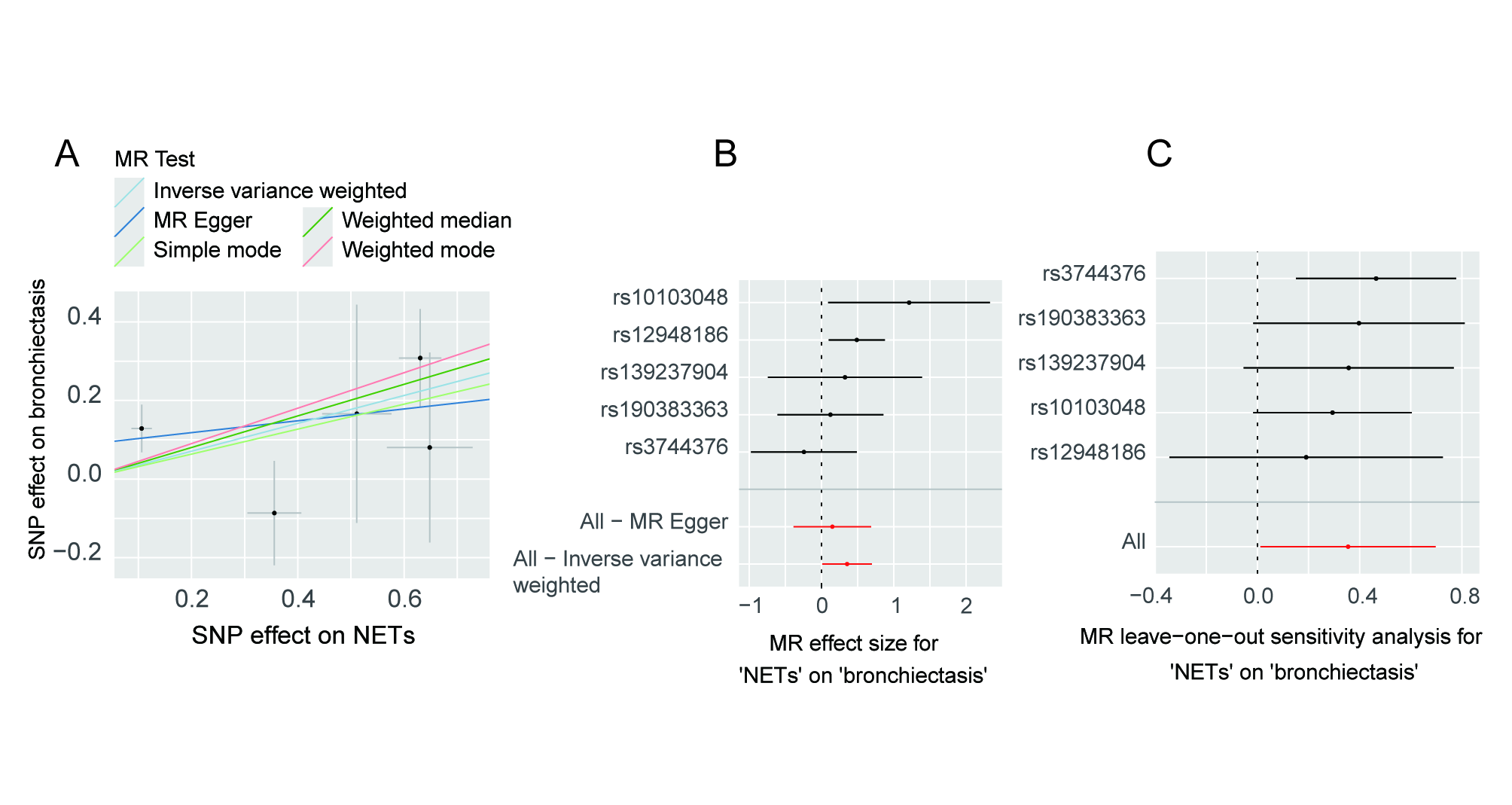

Supplement: Supplementary file 1 — Supplementary Material 1 [file 13568_2026_2012_MOESM1_ESM.tif]
