# Supplementary material for: Myeloperoxidase-DNA complex: a marker and combined target for Pseudomonas aeruginosa-associated bronchiectasis
Source: AMB Express. 2026 Jan 22;16:17. doi: 10.1186/s13568-026-02012-w (PMC12909637; doi:10.1186/s13568-026-02012-w)
Supplement: Supplementary file 2 — Supplementary Material 2 [file 13568_2026_2012_MOESM2_ESM.docx]

Supplementary table 1. Five SNPs were considered as IVs for the MPO-DNA complex.

| **SNP** | **effect_allele** | **other_allele** | **eaf** | **beta** | **SE** | **p value** |
| --- | --- | --- | --- | --- | --- | --- |
| rs10103048 | A | C | 0.393 | 0.106 | 0.019 | 2.20E-08 |
| rs12948186 | G | A | 0.057 | -0.630 | 0.040 | 2.03E-56 |
| rs139237904 | T | C | 0.021 | -0.511 | 0.065 | 4.44E-15 |
| rs190383363 | T | C | 0.014 | -0.648 | 0.081 | 9.19E-16 |
| rs3744376 | A | G | 0.034 | -0.356 | 0.051 | 2.22E-12 |

Annotation: eaf, expected average frequency; SE, standard error.
