# Supplementary material for: Myeloperoxidase-DNA complex: a marker and combined target for Pseudomonas aeruginosa-associated bronchiectasis
Source: AMB Express. 2026 Jan 22;16:17. doi: 10.1186/s13568-026-02012-w (PMC12909637; doi:10.1186/s13568-026-02012-w)
Supplement: Supplementary file 3 — Supplementary Material 3 [file 13568_2026_2012_MOESM3_ESM.docx]

Supplementary table 2. Results of MR analysis of MPO-DNA complex and 5 chronic respiratory diseases.

| **outcome** | **exposure** | **method** | **number of SNP** | **beta** | **SE** | **p value** | **OR** | **95%CI** | |
| --- | --- | --- | --- | --- | --- | --- | --- | --- | --- |
|  |  |  |  |  |  |  |  | **lower** | **upper** |
| bronchiectasis | NETs | MR Egger | 5 | 0.296 | 0.107 | 0.069 | 1.34 | 1.09 | 1.66 |
|  |  | Weighted median | 5 | 0.252 | 0.080 | 0.002 | 1.29 | 1.10 | 1.51 |
|  |  | Inverse variance weighted | 5 | 0.184 | 0.067 | 0.006 | 1.20 | 1.05 | 1.37 |
|  |  | Simple mode | 5 | 0.187 | 0.110 | 0.166 | 1.21 | 0.97 | 1.50 |
|  |  | Weighted mode | 5 | 0.224 | 0.088 | 0.064 | 1.25 | 1.05 | 1.49 |
| Chronic bronchitis | NETs | MR Egger | 5 | 0.209 | 0.203 | 0.380 | 1.23 | 0.83 | 1.84 |
|  |  | Weighted median | 5 | 0.013 | 0.104 | 0.898 | 1.01 | 0.83 | 1.24 |
|  |  | Inverse variance weighted | 5 | -0.061 | 0.156 | 0.696 | 0.94 | 0.69 | 1.28 |
|  |  | Simple mode | 5 | 0.106 | 0.147 | 0.512 | 1.11 | 0.83 | 1.48 |
|  |  | Weighted mode | 5 | 0.043 | 0.114 | 0.724 | 1.04 | 0.83 | 1.31 |
| IPF | NETs | MR Egger | 5 | -0.203 | 0.109 | 0.160 | 0.82 | 0.66 | 1.01 |
|  |  | Weighted median | 5 | -0.122 | 0.079 | 0.124 | 0.89 | 0.76 | 1.03 |
|  |  | Inverse variance weighted | 5 | -0.088 | 0.069 | 0.201 | 0.92 | 0.80 | 1.05 |
|  |  | Simple mode | 5 | -0.132 | 0.093 | 0.231 | 0.88 | 0.73 | 1.05 |
|  |  | Weighted mode | 5 | -0.130 | 0.086 | 0.207 | 0.88 | 0.74 | 1.04 |
| asthma | NETs | MR Egger | 5 | 0.021 | 0.029 | 0.517 | 1.02 | 0.97 | 1.08 |
|  |  | Weighted median | 5 | -0.003 | 0.021 | 0.874 | 1.00 | 0.96 | 1.04 |
|  |  | Inverse variance weighted | 5 | -0.011 | 0.018 | 0.548 | 0.99 | 0.95 | 1.03 |
|  |  | Simple mode | 5 | 0.011 | 0.030 | 0.739 | 1.01 | 0.95 | 1.07 |
|  |  | Weighted mode | 5 | -0.001 | 0.024 | 0.970 | 1.00 | 0.95 | 1.05 |
| COPD | NETs | MR Egger | 5 | 0.009 | 0.044 | 0.844 | 1.01 | 0.93 | 1.10 |
|  |  | Weighted median | 5 | -0.011 | 0.030 | 0.716 | 0.99 | 0.93 | 1.05 |
|  |  | Inverse variance weighted | 5 | -0.013 | 0.026 | 0.610 | 0.99 | 0.94 | 1.04 |
|  |  | Simple mode | 5 | -0.043 | 0.052 | 0.454 | 0.96 | 0.87 | 1.06 |
|  |  | Weighted mode | 5 | 0.028 | 0.031 | 0.419 | 1.03 | 0.97 | 1.09 |

Annotation: NETs, neutrophil extracellular traps; COPD, chronic obstructive pulmonary disease; IPF, idiopathic pulmonary fibrosis; SE, standard error; OR, odds ratio; 95%CI, 95% confidence interval.
