# Supplementary material for: Myeloperoxidase-DNA complex: a marker and combined target for Pseudomonas aeruginosa-associated bronchiectasis
Source: AMB Express. 2026 Jan 22;16:17. doi: 10.1186/s13568-026-02012-w (PMC12909637; doi:10.1186/s13568-026-02012-w)
Supplement: Supplementary file 4 — Supplementary Material 4 [file 13568_2026_2012_MOESM4_ESM.docx]

Supplementary table 3. Heterogeneity and horizontal pleiotropy of MR results.

| **outcome** | **exposure** | **pleiotropy** | | | **heterogeneity** | | | |
| --- | --- | --- | --- | --- | --- | --- | --- | --- |
|  |  | **Egger intercept** | | | **MR Egger** | | **Inverse variance weighted** | |
|  |  | **Egger intercept** | **SE** | **pval** | **Q** | **Q_pvalue** | **Q** | **Q_pvalue** |
| bronchiectasis | NETs | -0.052 | 0.038 | 0.268 | 0.935 | 0.817 | 2.772 | 0.597 |
| Chronic bronchitis | NETs | -0.126 | 0.074 | 0.186 | 5.918 | 0.116 | 11.688 | 0.020 |
| COPD | NETs | -0.011 | 0.016 | 0.556 | 3.922 | 0.270 | 4.492 | 0.343 |
| IPF | NETs | 0.054 | 0.040 | 0.268 | 0.822 | 0.844 | 2.666 | 0.615 |
| asthma | NETs | -0.015 | 0.010 | 0.247 | 2.060 | 0.560 | 4.115 | 0.391 |

Annotation: NETs, neutrophil extracellular traps; COPD, chronic obstructive pulmonary disease; IPF, idiopathic pulmonary fibrosis; SE, standard error.
