# Supplementary material for: Myeloperoxidase-DNA complex: a marker and combined target for Pseudomonas aeruginosa-associated bronchiectasis
Source: AMB Express. 2026 Jan 22;16:17. doi: 10.1186/s13568-026-02012-w (PMC12909637; doi:10.1186/s13568-026-02012-w)
Supplement: Supplementary file 5 — Supplementary Material 5 [file 13568_2026_2012_MOESM5_ESM.docx]

Supplementary table 4. Reverse MR analysis results of MPO-DNA complex and 5 chronic respiratory diseases.

| **outcome** | **exposure** | **method** | **number of SNP** | **b** | **SE** | **p value** | **OR** | **95%CI** | | **MR Egger intercept.P** | **Heterogeneity** | |
| --- | --- | --- | --- | --- | --- | --- | --- | --- | --- | --- | --- | --- |
|  |  |  |  |  |  |  |  | **lower** | **upper** |  | **Q** | **P** |
| NETs | finngen-bronchiectasis | MR Egger | 14 | -0.003 | 0.020 | 0.871 | 1.00 | 0.96 | 1.04 |  | 16.519 | 0.169 |
| NETs | finngen-bronchiectasis | Weighted median | 14 | -0.002 | 0.018 | 0.927 | 1.00 | 0.96 | 1.03 |  |  |  |
| NETs | finngen-bronchiectasis | Inverse variance weighted | 14 | -0.012 | 0.018 | 0.487 | 0.99 | 0.95 | 1.02 | 0.331 | 17.930 | 0.160 |
| NETs | finngen-bronchiectasis | Simple mode | 14 | -0.028 | 0.064 | 0.665 | 0.97 | 0.86 | 1.10 |  |  |  |
| NETs | finngen-bronchiectasis | Weighted mode | 14 | -0.001 | 0.016 | 0.942 | 1.00 | 0.97 | 1.03 |  |  |  |
| NETs | GCST90044075-bronchiectasis | MR Egger | 11 | -0.026 | 0.043 | 0.558 | 0.97 | 0.90 | 1.06 |  | 8.313 | 0.503 |
| NETs | GCST90044075-bronchiectasis | Weighted median | 11 | 0.021 | 0.025 | 0.402 | 1.02 | 0.97 | 1.07 |  |  |  |
| NETs | GCST90044075-bronchiectasis | Inverse variance weighted | 11 | 0.009 | 0.019 | 0.646 | 1.01 | 0.97 | 1.05 | 0.268 | 9.127 | 0.520 |
| NETs | GCST90044075-bronchiectasis | Simple mode | 11 | 0.025 | 0.038 | 0.526 | 1.03 | 0.95 | 1.11 |  |  |  |
| NETs | GCST90044075-bronchiectasis | Weighted mode | 11 | 0.014 | 0.035 | 0.693 | 1.01 | 0.95 | 1.09 |  |  |  |

Annotation: NETs, neutrophil extracellular traps; COPD, chronic obstructive pulmonary disease; IPF, idiopathic pulmonary fibrosis; b, beta value; SE, standard error; OR, odds ratio; 95%CI, 95% confidence interval.
