# Supplementary material for: Myeloperoxidase-DNA complex: a marker and combined target for Pseudomonas aeruginosa-associated bronchiectasis
Source: AMB Express. 2026 Jan 22;16:17. doi: 10.1186/s13568-026-02012-w (PMC12909637; doi:10.1186/s13568-026-02012-w)
Supplement: Supplementary file 6 — Supplementary Material 6 [file 13568_2026_2012_MOESM6_ESM.docx]

|  | **Sham Group** | | | **Model Group** | | | **AZD5904 Group** | | | **DNase I Group** | | | **AZD5904 + DNase I Group** | | |
| --- | --- | --- | --- | --- | --- | --- | --- | --- | --- | --- | --- | --- | --- | --- | --- |
| Sample number | 1 | 2 | 3 | 1 | 2 | 3 | 1 | 2 | 3 | 1 | 2 | 3 | 1 | 2 | 3 |
| Columnar ciliated epithelium (A) | 0 | 0 | 1 | 1 | 1 | 2 | 0 | 0 | 1 | 0 | 0 | 1 | 0 | 0 | 0 |
| Gland cells (B) | 0 | 0 | 0 | 1 | 1 | 1 | 0 | 1 | 0 | 0 | 1 | 0 | 1 | 1 | 0 |
| Intraluminal secretions (C) | 1 | 0 | 1 | 1 | 1 | 1 | 1 | 1 | 1 | 1 | 0 | 0 | 0 | 0 | 1 |
| Inflammatory cell infiltration (D) | 0 | 0 | 0 | 3 | 3 | 3 | 1 | 1 | 1 | 1 | 1 | 1 | 0 | 1 | 1 |
| Total score | 1 | 0 | 4 | 8 | 8 | 11 | 2 | 3 | 5 | 2 | 2 | 4 | 1 | 2 | 2 |

Supplementary table 5. Tracheobronchial histopathological score of each sample.

Annotation: Total score = 3A + B + D + C.
